# Ideal-observer model of human sound localization of sources with unknown spectrum

**DOI:** 10.1038/s41598-025-91001-3

**Published:** 2025-03-01

**Authors:** Jonas Reijniers, Glen McLachlan, Bart Partoens, Herbert Peremans

**Affiliations:** 1 Department of Engineering Management, Universiteit Antwerpen, 2000 Antwerp, Belgium; 2 Department of Physics, Universiteit Antwerpen, 2020 Antwerpen, Belgium

**Keywords:** ideal-observer, sound localization, information theory, Bayes, Psychology, Human behaviour, Computational biology and bioinformatics, Computational models

## Abstract

Localization of a sound source is in essence the act of decoding the directional information with which the sound was endowed by the head and ears upon measurement by the cochlea. Yet, as the source’s directional signature is conflated with the spectral characteristics of the source and the latter is often not known to the listener, this directional signature may be obscured, hampering localization. Current localization models generally avoid this problem by considering sources whose spectrum is known to the listener. In this paper, we investigate how an ideal-observer would deal with this uncertainty of the source: by means of a prior on the source spectrum built from previous experiences. To this end, an ecologically valid prior was constructed from databases of environmental sounds and speech. Incorporation of this prior allowed to explain the results of a localization experiment in which the stimulus was varied, without *any* parameter fitting. It was shown that if the spectrum of the source deviates too much from those of real-world environments, this results in localization errors, because the source does not fit the prior used by the listener. Moreover, it seems that the binaural spectral gradient contains the relevant spectral information and that the ipsilateral side has more weight in the decision. We could not corroborate the experimental indication that only the positive spectral gradient values are used for localization. Finally, the model including the ecologically valid prior was also better in explaining the experimental data on localization of invariably flat spectrum stimuli, allowing for the possibility that human listeners may rather use a multi-purpose than a situation-specific spectral prior.

## Introduction

Localization of a sound source requires a listener to decode the directional information that is embedded in the sounds recorded by both ears^1–3^. Evolution has molded our hearing system such that it endows the sounds picked up by both ears with cues bearing information on the direction of the source^4^. Our ears are positioned on opposite sides of the head, resulting in interaural time differences (ITD) and interaural level differences (ILD), which provide information on the lateral angle of the source. This restricts the source to lie on one of the so-called ‘cones of confusion’^5^. In addition, the morphology of the ears has been adapted such that their directional filtering endows the spectrum with cues on the polar angle of the source^1^. Yet, even with these directional cues, it is not straightforward to solve the inverse problem and infer from which direction the sound came^2^. Considerable research effort has been spent on understanding localization performance under different listening conditions, such as altered directional filtering^6,7^, source spectra^8,9^, room reflections^10,11^, or playback methods^12–15^. Dynamic cues, e.g. due to head or source movement, also play an important role, especially for resolving front/back confusions^16^. In this paper we restrict ourselves to localization of short broadband stimuli in absence of dynamic cues (neither source, nor head movement).

The accuracy of sound source localization is fundamentally limited by the precision with which the hearing system can encode the observed time differences and the spectral detail as this injects uncertainty into the solution of the inverse problem^2^. Consequently, a great deal of research has focused on understanding localization performance in terms of the ITD, ILD and spectral content given the limitations of the hearing system, both with regards to the type of errors (front/back confusions, upper/lower confusions) as the accuracy, precision and bias of sound localization^17^.

However, most of this empirical research as well as the subsequent modelling^2,3^ has focused on a particular simplification, i.e., studying localization of sources whose spectrum was known to the observer. Indeed, in most psycho-acoustic experiments the (magnitude of the) spectrum of the stimulus does not change between trials. Yet, this assumption does not hold in real-life situations, where the subject is confronted with a large variety of different sounds of which the spectrum is often unknown to the listener. As a consequence, the directional spectral signature is conflated with a variable and unknown spectrum of the source adding to the ill-posed nature of the inverse problem and making it even harder to solve^2,18^.

It remains to be determined how human listeners (and, by extension, other mammals) solve this problem. Is the auditory system able to distinguish between spectral features of the source and directional cues, and if so, how is this achieved^19^? Does the brain expect a broad, flat source spectrum^20^, or does it expect that the spectrum has a locally constant slope to distinguish between the spectral features^21^? Is the entire spectral profile best suited for this task, or does the brain focus on specific spectral features, such as notches or peaks^22^? Indeed, one could conjecture that the spectral filtering by the ear has evolved such that the resulting peaks/notches differ as much as possible from the variations imposed by the source. Ultimately, disentangling the directional cues from the spectral cues inherent to the source requires a strategy which is adapted to the environment of the listener and takes into account the statistics of the ensemble of sound sources that she is confronted with. Traer and McDermott^23^ showed that listeners could indeed disentangle the source spectrum from the reverberation (separating the source from the environment), but only if the reverberation characteristics did not deviate too much from those of real-world environments. Francl and McDermott^18^ equipped a neural network with human ears and trained it to localize sounds in realistic conditions (natural sources, noise, reverberation) and showed that training in natural conditions was essential to reproduce human localization performance. Both studies suggest that the listener makes use of expectations about natural source spectra, noise, and reverberation. However, these studies do not allow explicit manipulation of these expectations to study their impact on the localization process.

Hence, in this paper we follow a regularization approach to the ill-posed localization problem based on Bayesian reasoning that enables us to implement these expectations as an explicitly defined prior. Moreover, such an approach is in general more transparent, as it allows one to track the information transfer and trace the origin of certain erroneous localization trials. To this end, we adapt the ideal-observer model that we proposed previously^3^. This model reveals the best theoretically possible localization performance that can be achieved by an optimal combination of all available information, given the limitations imposed by the precision of the human auditory system as estimated from psychoacoustic discrimination experiments. Previously, we limited ourselves to localization when the source spectrum was purportedly known to the listener, e.g. because the subject was confronted with the same stimulus throughout the experiment. We modelled this situation by using an ad hoc prior, an ‘educated guess’. Here, we adapt this source prior such that it operates optimally in the context of the large variability of sounds that listeners are confronted with in a real-life setting. We consider a prior for the source spectrum that is built from two different databases of sounds that are deemed relevant to human listeners: one with environmental sounds^24^ and one with human speech^25^. The rationale is that these databases reflect the spectral variations of the ensemble of sound stimuli that human listeners are confronted with in real life, resulting in a prior which is *ecologically valid*^26^. Note that, with this estimate of the source spectrum prior, all parameters of the ideal-observer model are based on data that are unrelated to the localization performance that we are trying to understand. This makes our approach essentially different from the one taken by Baumgartner et al.^27^ and Barumerli et al.^28^, where the model parameters are fitted to reproduce individual subject’s localization data.

**Fig. 1:**
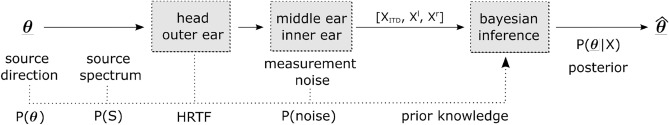
Schematic representation of the ideal-observer model.

In addition to a novel prior, we also adapt the original ideal-observer model to test for different acoustic features as input. The previous model relied on the ITD and the spectral magnitude. Yet, there are behavioural^29^ and neuro-physiological^30^ indications that other spectral features may be used for sound localization^27,28^. Here, we consider (1) the binaural spectral gradient (SG), defined as the slope (derivative) of the spectral log-magnitude along the frequency axis; (2) the ipsilateral SG, where only the SG of the ipsilateral ear is used for localization, and (3) the positive SG, which consists only of those spectral components for which the SG is positive. The model was tested for each different acoustic feature against the experimental localization data of Macpherson et al.^31^, as was also done in Refs.^27,28^. In their experimental study, subjects were confronted with stimuli whose spectrum was modulated with a sinusoidal ripple along the frequency axis, which varied between trials. To assess the implications of an ecologically valid prior for localization of stimuli with a flat spectrum, we also revisited the meta-analysis of localization studies with broadband flat spectrum stimuli^17^ and investigated whether a general prior may explain/resolve some of the discrepancies that were encountered previously^3^.

## Methods

### Ideal-observer model

Previously we have proposed an ideal-observer model for sound localization of short broadband sounds in the far field.^3,32^. It predicts the subjects’ performance under the assumption that subjects make optimal use of the available information. For clarity, we only describe the model in very general terms here. A detailed derivation and description of the model is included in the Supplementary Information.

The model views sound localization as an encoding/decoding process, where direction dependent filtering by the head and ears endows the acoustic input with certain directional cues, see Fig. 1. The observer receives an acoustic input $\textbf{X}$ and wants to infer $P(\varvec{\theta }|\textbf{X})$, the probability that, given the observed acoustic input, the sound originates from direction $\varvec{\theta }$. To this end, the ideal-observer makes maximal use of prior knowledge about the source direction $P(\varvec{\theta })$, about the source spectrum $P(\textbf{S})$ (based on previously encountered source spectra), about the directional filtering by head and ears (HRTF), and about the measurement noise due to the limited precision of the hearing system $P(\text {noise})$. Using Bayes’ rule, the prior $P(\varvec{\theta })$ can then be updated, taking into account the likelihood of the acoustic input $P(\textbf{X}|\varvec{\theta })$, to the posterior

1 $$\begin{aligned} P(\varvec{\theta }|\textbf{X}) = \frac{1}{C}P(\textbf{X}|\varvec{\theta }) P(\varvec{\theta }) \end{aligned}$$

with *C* a normalization constant such that $\int P(\varvec{\theta }|\textbf{X}) d\varvec{\theta } =1$. There are indications that human observers use a non-uniform spatial prior $P(\varvec{\theta })$ with a bias towards the horizontal plane reflecting the higher probability/relevance of sources at these directions^33^. However, to keep model complexity to a minimum, we consider a uniform prior probability, so the problem reduces to the calculation of the likelihood function $P(\textbf{X}|\varvec{\theta })$.

If we consider the ITD supplemented with the log-magnitudes of the left and right ear spectral input $\textbf{X}^l$ and $\textbf{X}^r$ as the relevant acoustic input

2 $$\begin{aligned} \textbf{X}= [X_\text {ITD}, \textbf{X}^l, \textbf{X}^r], \end{aligned}$$

One can write the likelihood function as a multivariate normal distribution,

3 $$\begin{aligned} P(\textbf{X}|\varvec{\theta }) \sim \mathcal {N}(\textbf{T}(\varvec{\theta }),\varvec{\Sigma }), \end{aligned}$$

centered around a direction-dependent template $\textbf{T}(\varvec{\theta })$ with a covariance matrix $\varvec{\Sigma }$ which is independent of $\theta$ and the acoustic input $\textbf{X}$^3^. This covariance matrix partly reflects the uncertainty due to measurement noise, $P(\text {noise})$, and partly the uncertainty on the source spectrum, $P(\textbf{S})$. The directional-dependent template contains information both about the directional filtering (HRTF) and the most likely source spectrum according to the source prior $P(\textbf{S})$.

Note that the likelihood function is modeled as a multivariate distribution. This parameterization of the likelihood function simplifies things greatly, especially if both the source prior $P(\textbf{S})$ and the noise prior $P(\text {noise})$ can be modeled as multivariate distributions. Indeed, the product of two multivariate distributions is again a multivariate distribution of which the covariance matrix can be easily obtained from the covariance matrices of the respective distributions.

Yet, such a parameterization of the priors requires that the acoustic information is transformed. Therefor, the ITD and spectral magnitude are expressed in units of just-noticeable differences (jnd’s) as measured in psychoacoustic discrimination experiments, such that the ‘measurement noise’ accounting for the limited precision of the auditory system can be modeled as Gaussian additive noise with a variance that is *independent* of the sensory input (ITD and spectral magnitude). As a result, one only has to consider a single covariance matrix $\varvec{\Sigma }$ for the prior in Eq. (3), irrespective of the angle $\varvec{\theta }$. With regards to the spectral magnitude, this means considering the log-magnitude (in dB); for the ITD this entails a slightly different nonlinear transformation, see Supplementary Information. In addition, the spectral magnitude is defined at equivalent rectangular bandwidth (ERB) centre frequencies in order to have independent frequency channels^34^. This results in 30 frequency channels with centre frequencies between 300 Hz and 12 kHz. The broadband stimulus is considered above the hearing threshold (or noise level) for all channels.

It is a major strength of this approach that the model parameters (i.e., the parts of the covariance matrix) that account for uncertainty due to measurement noise can be deduced from psycho-acoustic discrimination experiments that do not involve sound localization^3^. However, for the part of the covariance matrix that accounts for the uncertainty (prior knowledge) about the source spectrum, we did not find psycho-acoustic experiments providing the information we need. For this reason, we put forward an ad hoc prior in our previous work^3^. In this paper, we follow a different approach and deduce the (mean and covariance matrix of the multivariate) source prior from another ‘independent’ source: from two databases of environmental sounds and speech. Hence, there are no free parameters left in the proposed model.

### Spectral features used for localization

The original ideal-observer model described above assumes that both left and right log-magnitude spectra are used for localization, and have equal weight in the estimation process. Yet, as mentioned in the introduction, there are indications that not all spectral information may be used, but only specific spectral features. Below, we describe how we adapt the inputs to the ideal-observer model so as to better correspond with the hypotheses about the human localization process that have been suggested as alternatives.

#### Binaural spectral gradient

Instead of the acoustic input shown in Eq. (2), we consider the following input:

4 $$\begin{aligned} \textbf{X}=[X_\text {ITD},X_\text {ILD},\textbf{dX}^l,\textbf{dX}^r], \end{aligned}$$

i.e., the ITD is supplemented with the broadband ILD, here defined as

5 $$\begin{aligned} X_\text {ILD} = \frac{1}{N}\sum _{i=1}^N (\textbf{X}^l_i - \textbf{X} ^r_i). \end{aligned}$$

and the left and right SGs which are defined as the set of differences between neighbouring frequency channels

6 $$\begin{aligned} \textbf{dX}_i^{l/r} = \textbf{X}_i^{l/r}-\textbf{X}_{i-1}^{l/r}. \end{aligned}$$

**Fig. 2:**
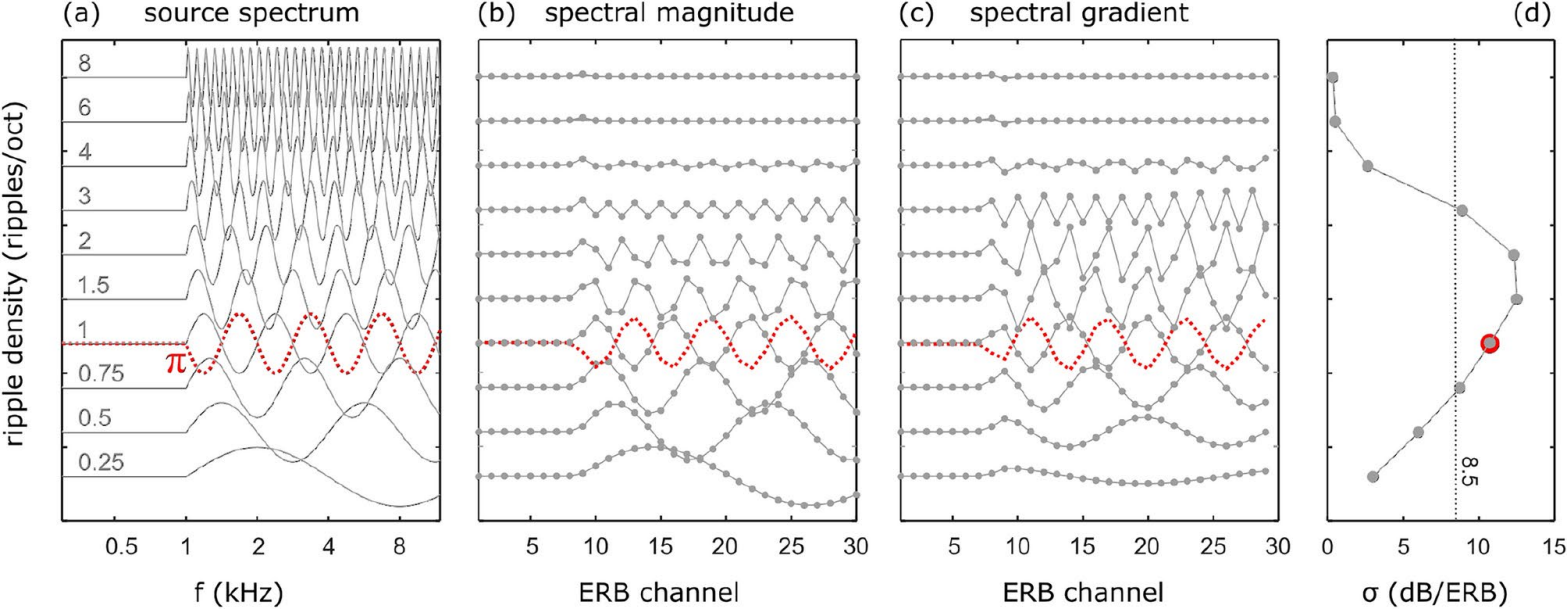
(**a**) Ripple spectrum for different ripple densities; (**b**) the spectral magnitude in each of the frequency channels; (**c**) the gradient of the spectral magnitude; (**d**) the standard deviation of the SG, as function of the ripple density. The dotted red curves show the ripple spectrum in case of phase $\phi =\pi$, all other curves have phase $\phi =0$.

An example of the SG representation is shown for the source spectrum in Fig. 2c: if the spectral log-magnitudes of rippled-spectrum stimuli (Fig. 2a) are subsampled at discrete ERB frequencies (Fig. 2b), and one takes the derivative along the ERB-axis, one arrives at the spectral gradient SG (Fig. 2c). Since this transformation from spectral log-magnitude to SG is linear, see Eqs. (5) and (6), the likelihood function is again a multivariate normal distribution, with direction-dependent templates and a covariance matrix that can be derived from those used previously in Eq. (3). It can be shown that this representation contains exactly the same directional information as the original binaural log-magnitude representation, if the overall sound level carries no directional information, as is indeed the case.

#### Positive spectral gradient

Here, we consider the case where only the positive SG values^27,28^ are used by the direction estimation process (again supplemented with ITD and ILD). While there are indications that only the positive SG is relayed to the dorsal cochlear nucleus for polar angle estimation^30^, the evidence is not very strong, since this was not observed in guinea pigs^35^. Note that in this case there is a loss of information as this transformation of the log-magnitude spectra can not be inverted. Again, the likelihood function can be written as a multivariate normal distribution, with angular templates and covariance matrix that can be derived from those used in Eq. (3).

#### Ipsilateral spectral gradient

Here, we consider another transformation of the SG that also results in information loss, i.e. we limit the spectral input to the SG of the presumed ipsilateral ear. Again there are a number of studies that demonstrated that the spectral cues from the ear contralateral to the target have a decreasing weight in the direction estimation process becoming negligible for targets sufficiently displaced from the midline^36,37^. Considering only the ipsilateral SG can be viewed as a limiting case. The true performance should be somewhere in between that obtained using the binaural SG (upper limit) and that obtained with the ipsilateral SG (lower limit). For simplicity, we base the decision on which SG is used as input to the model, $\textbf{dX}^l$ or $\textbf{dX}^r$, solely on the sign of the measured ITD. Since only a single monaural SG is considered, the correlation between left and right source spectrum can no longer be exploited. Note, though, that the source spectrum correlation is still present and possibly exploited through the ILD. Hence, both the ITD and ILD remain truly binaural cues.

#### Interaural spectral difference

As a baseline, we also consider the case where the observer assumes no prior knowledge on the source spectrum at all, i.e., every source spectrum is equally probable. This situation is identical to using only ITD and the Interaural Spectral Difference $\text {ISD} = \textbf{X}^l - \textbf{X} ^r$ as acoustic input, as already discussed in our previous work^3^. Indeed, since both ears experience an identical source spectrum, subtraction of both log-magnitude spectra removes the source spectrum from the acoustic input, and hence this representation only contains information that is independent of the source spectrum.

### Source prior calculation

To estimate the prior of the source spectra/SGs, we used two different freely available online databases.

CREMA-D^25^ is a data set of 7442 original clips from 48 male and 43 female actors between the ages of 20 and 74 coming from a variety of races and ethnicities (African America, Asian, Caucasian, Hispanic, and Unspecified). Actors spoke from a selection of 12 sentences. The sentences were presented using one of six different emotions (Anger, Disgust, Fear, Happy, Neutral, and Sad) and four different emotion levels (Low, Medium, High, and Unspecified).

ESC-50^24^ is a collection of 2000 environmental audio recordings suitable for benchmarking methods of environmental sound classification. The dataset consists of 5-second-long recordings organized into 50 semantical classes.

Each of the sound files of the database were chopped up in intervals of 0.2 s and for each of these intervals, the source log-magnitude spectrum was expressed as function of the ERB centre frequencies. The resulting spectra were pooled in a single dataset, from which the average source spectrum and the covariance matrix were calculated.

### Benchmark experiments

To evaluate the different models, we use the experimental data obtained by Macpherson and Middlebrooks^31^. In a series of experiments, the subjects had to localize short (250 ms) broadband stimuli: random-phase noise bursts of which the spectrum was varied between consecutive trials. Source positions varied randomly between 25 positions in the front and 25 in the rear, distributed approximately uniformly within a range of $30^\circ$ from the midsagittal plane and $60^\circ$ above and below the horizontal plane. Hence, the experiment focused on localization of sound sources close to the midsagittal plane.

The subjects had no prior knowledge on the stimulus spectrum: in each consecutive trial, stimuli with a flat spectrum were alternated with stimuli with a modulated spectrum; the spectral shape of these stimuli was modulated as a cosine function of the log-frequency, see Fig. 2a. The modulation was characterized by three parameters: the peak-to-through amplitude *A* (in dB), the ripple density $\rho$ (ripples/octave) and the ripple phase $\phi$. The impact of these parameters on the localization performance was investigated in three separate experiments.

Localization performance was quantified by the polar ‘error rate’, defined as the proportion of responses exhibiting polar errors that exceed $45^\circ$ (the exact definition is a bit more subtle; see Macpherson et al.^31^). This threshold was chosen to encompass the scatter in the flat-frequency responses and to arrive at a robust performance measure. In total six subjects participated in the experiment. Because of the rather large variability in the localization performance between these six individuals we average error rate over all participants.

We also investigate how the different versions of the model compare with human listeners in case the test subjects were confronted with an invariably flat spectrum stimulus. To this end, we use a meta-analysis of localization trials that have been conducted over several years in four different laboratories^17^.

### Monte-Carlo simulations

Monte-Carlo simulations were ran to reproduce the experimental results. All model simulations are done for 20 different HRTFs taken from the online ARI database (20 first ones in the dataset)^38^. These HRTFs were spatially resampled to 2000 directions distributed uniformly over the sphere (using spherical harmonics interpolation with Tikhonov regularization)^39^. Directions with elevation below $45^\circ$ were omitted since the HRTFs were not measured in this area, resulting in a total of 1708 directions. To calculate the error rates, we did not restrict ourselves to the 50 directions that were sampled in the experiment, but included all (599) directions that were within the range of $30^\circ$ from the midsagittal plane and $60^\circ$ above and below the horizontal plane. For each HRTF, each of these directions was virtually presented once, i.e., a different noise realisation was generated for each trial. A noise realisation was generated by sampling randomly from a multivariate distribution with the model’s proper measurement covariance matrix $\varvec{\Sigma }$ whereby we set the part of $\varvec{\Sigma }$ describing the uncertainty about the source spectrum equal to zero. For each trial, the *maximum posterior* criterion was used to arrive at an estimate of the source direction $\hat{\varvec{\theta }}$. The simulated error rates of the different subjects are all averaged into a single value with a low variance (std $<2\%$ of mean value) due to the large total number of trials. Hence, we only show the average simulated error rate and do not discuss its variance.

## Results

### Source prior

**Fig. 3:**
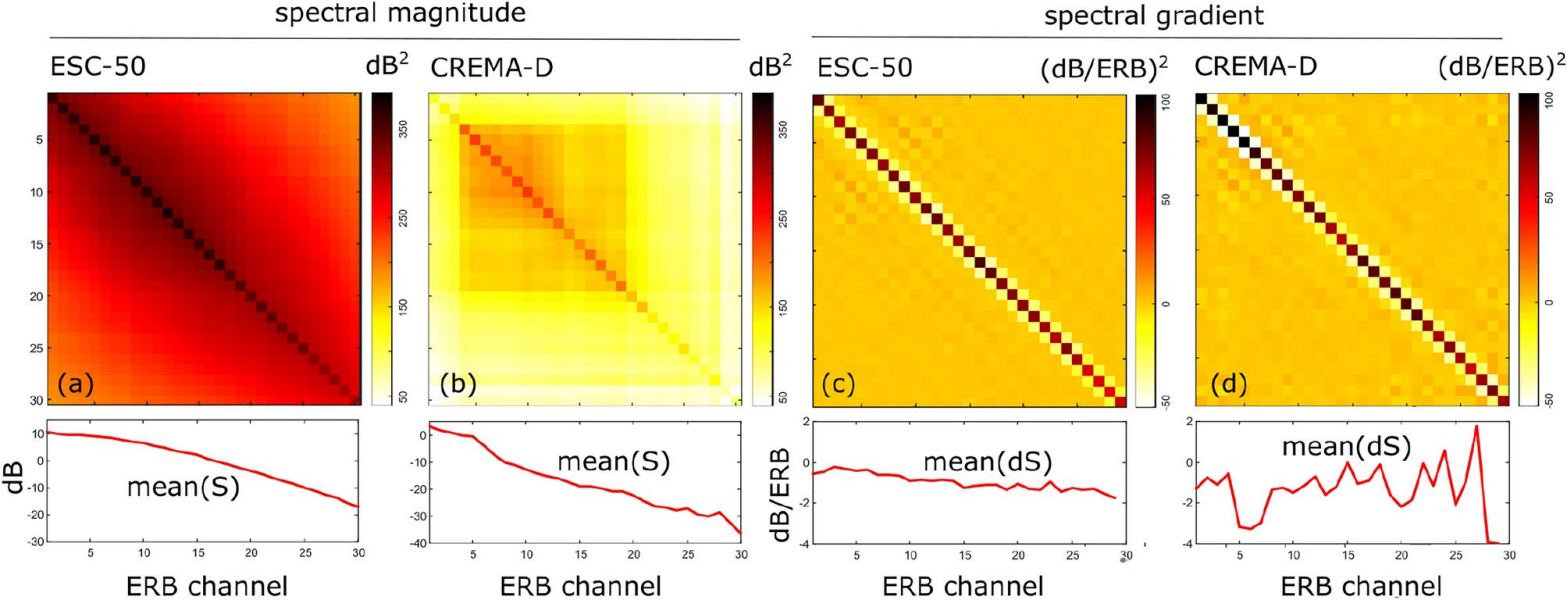
The source prior covariance matrix (top) and mean spectral magnitude (bottom) as built from (**a**) the ESC-50 and (**b**) the CREMA-D database; (**c**, **d**) the same, but in case the spectral gradient is considered.

In Fig. 3a and b we have plotted the covariance matrices for the two databases, with their respective mean spectral magnitude. The covariance and mean spectral magnitude fully describe the source prior $p(\textbf{S})$ if it is parameterized as a multivariate distribution to calculate the likelihood function in Eq. (3).

The two covariance matrices are clearly different, yet they also display similarities: both covariance matrices exhibit large variances on the diagonal and a nonzero covariance between frequency channels, which decreases as the channels are farther apart. Overall, the channels have a large non-zero background covariance, indicating large sound level differences between different acoustic stimuli. The mean spectral magnitude contains most energy at lower frequencies (ERB channel numbers) and decreases with increasing frequency. The overall decrease at 12 kHz is approximately 30 dB and 40 dB for the two databases.

Inspection of the covariance matrices of the SG in Fig. 3c and d reveals a more striking similarity between both matrices: both are mainly tridiagonal, with rather large variances on the main diagonal and a negative upper and lower diagonal, reflecting negative correlation between nearby gradient coefficients. The mean SG varies over a much smaller range (approximately 2 dB and 3 dB), compared to the mean spectral magnitude.

In the following, all simulations are carried out using the prior built from the ESC-50 dataset (environmental sounds), since the results are very similar in case of the prior built from the CREMA-D database, see Supplementary Information.

### Ripple density

**Fig. 4:**
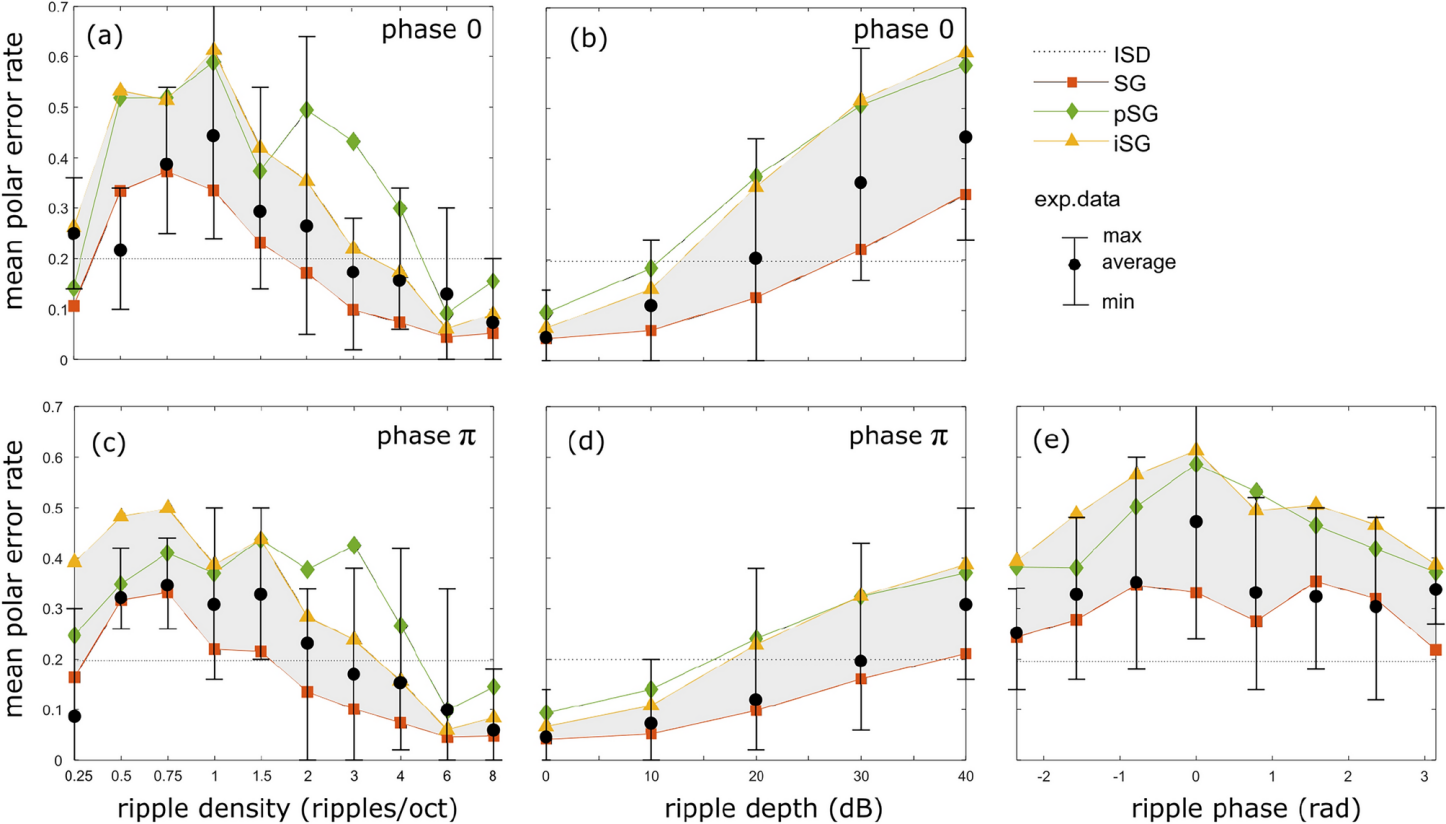
The average polar error rate (**a**, **c**) as function of ripple density in case of phase 0 and phase $\pi$ (40dB ripple depth); (**b**, **d**) as function of ripple depth for phase 0 and $\pi$ (1 ripple/oct); (**e**) as function of ripple phase (1 ripple/oct, 40dB ripple depth). The experimental results are shown in black (the whiskers mark the minimal and maximal error rate of the 6 subjects); the different model predictions considering the different features are shown in color.

The polar error rate is shown in Fig. 4a as function of the ripple density (in case of ripple phase $\phi =0$ and a ripple depth of 40 dB). The experimental error rate is rather small at low ripple densities, grows with increasing ripple density to attain a peak around 1–2 ripples/octave and again decreases at higher ripple densities. In case the listener would use no prior at all (ISD), the error rate invariably equals $\approx 0.2$ irrespective of the ripple density. If a prior on the SG is included, the same overall pattern is observed as in experiment, although the simulated error rates are consistently smaller. The error rates increase if only the ipsilateral (iSG) or the positive (pSG) SG are considered, yet the latter curve deviates from that observed experimentally, especially for higher ripple densities.

### Ripple depth

The experimental polar error rate (in case of ripple phase $\phi =0$ and ripple density of 1 ripple/octave) increases almost linearly with increasing ripple depth, see Fig. 4b. All models show a similar behaviour (except for the ISD model), though simulated error rates are too small in case of the SG, and too large in case of ipsilateral and positive SG input.

**Fig. 5:**
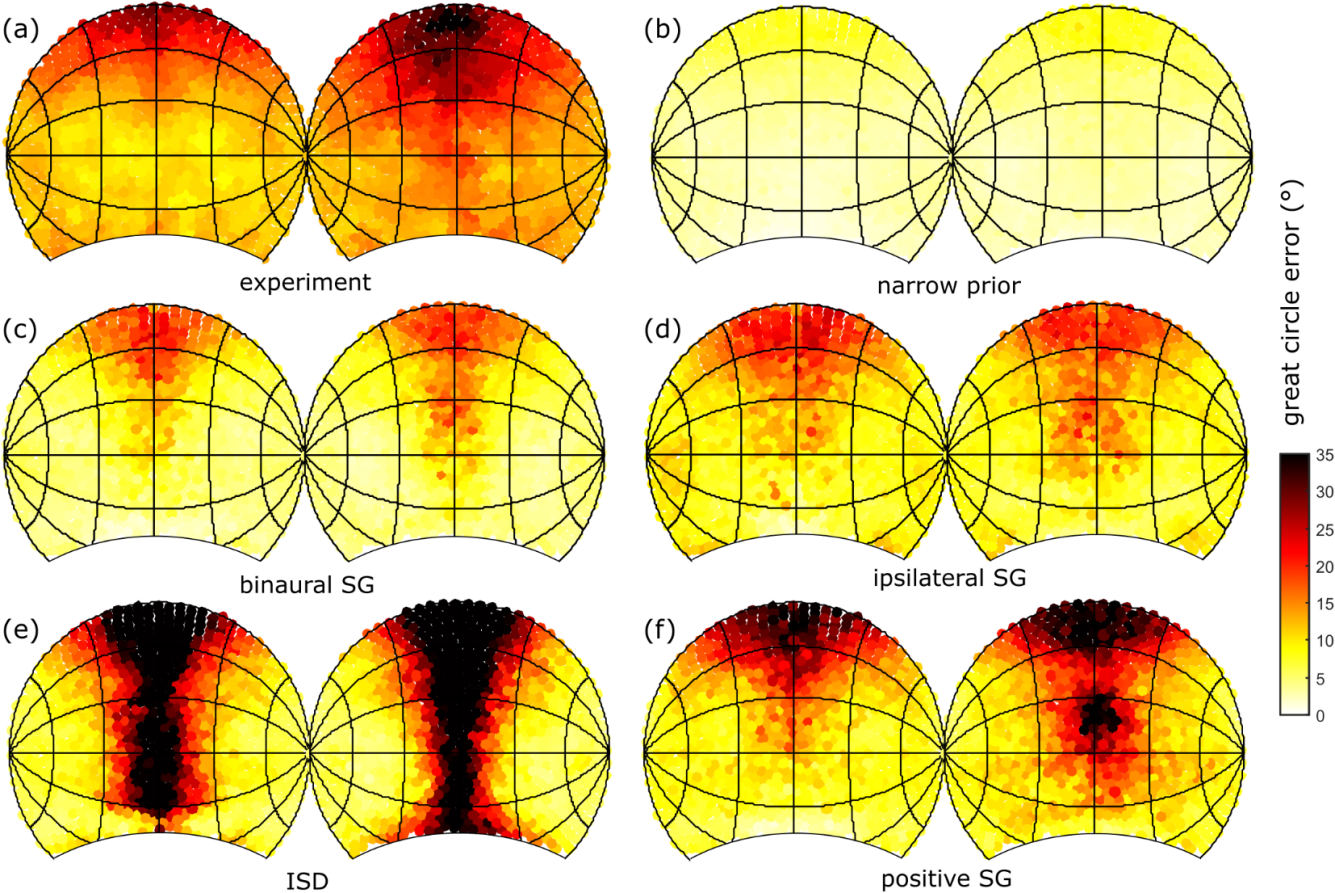
The great circle error for localization of a flat source stimulus (**a**) as measured experimentally^17^ and as predicted by the ideal-observer model assuming: (**b**) no source prior (ISD), (**c**) a narrow ad hoc prior as in Reijniers et al. ^3^, the ESC-50 prior in case of (**d**) binaural SG, (**e**) ipsilateral SG, (**f**) the positive SG input.

### Ripple phase

If we consider the same graphs in case of ripple phase $\pi$, see Fig. 4c and d, we observe overall the same pattern, except that the error rates are slightly lower compared to those in case of $\phi =0$. This behaviour is observed both in experiment and simulation. The difference is most pronounced for a ripple density of 1 ripple/octave. This is also obvious from Fig. 4e, where the error rates are shown (in case of 40dB ripple depth and a ripple density of 1 ripple/octave) as function of ripple phase. The experimental error rate depends (slightly) on the phase and is on average highest in case of phase 0. In case of the SG input, we do not observe this behaviour as the error rate remains rather constant, yet, in case of positive and ipsilateral SG input, the error rate does indeed attain the peak at phase 0, which is also observed in experiment.

### Flat spectrum

The great circle error is shown in Fig. 5 in case of localization of a flat spectrum sound source. The angular errors obtained in the meta-analyses^17^ are shown in Fig. 5a: the average error ranges between $10^\circ$ and $35^\circ$, the errors being largest on top and larger in the rear compared to the front. In Fig. 5b, the average error is shown in case of the ideal-observer model when using no prior at all on the source spectrum (ISD). In this case, angular errors are large for directions close to the midsagittal plane, an observation that is absent in experiment. Assuming the ad hoc narrow prior from Reijniers et al.. ^3^, the resulting error shows the same overall pattern as in Fig. 5a, yet it is considerably smaller than observed experimentally ($<12^\circ$). Assuming the new generic prior (binaural/ipsilateral SG) increases the errors by a factor two, the largest errors occurring in case of the ipsilateral SG input, lifting the errors towards the levels observed in experiment, see Fig. 5d and e. The simulated errors are also larger on top, yet they are not substantially larger in the rear compared to the front. Finally, when considering only the positive SG, see Fig. 5f, we again obtain errors that exceed those observed in experiment.

## Discussion

Assuming that the overall level of the spectrum carries no directional information, using the binaural SG and ILD cues instead of the binaural log-magnitude spectra is equivalent in terms of information provided to the ideal-observer model. However, our analysis shows that the SG representation is not only more compact (by removing the overall sound level), but it is also more instructive with regards to the similarities of the covariances obtained from different databases. Figure 3 shows that the covariance matrices for both databases are quite different in terms of the spectral magnitudes, yet their covariance matrices are much more alike when the spectral information is rewritten in terms of the SG. Moreover, the spectral magnitude representation includes information about the sound level which is irrelevant for localization and which is removed in case of the SG. This insensitivity of the SG distribution for the specific set of ecologically valid stimuli suggests that in real-life situations listeners would benefit from exploiting SG cues represented by a prior with a covariance matrix very similar to the ones shown in Fig. 3c and d as this prior would be generic, i.e., not context dependent.

Before discussing the impact of a particular source prior on localization, it is instructive to inspect the performance that would be achieved, if the subject were to use no prior information about the source spectrum at all by relying on the binaural cues ISD and ITD only. In this case, our model predicts (dotted curve in Fig. 4) that the polar error rate is independent of the source spectrum (as expected) and always equals $\approx 0.2$, irrespective of the ripple parameters. This behaviour is clearly not in agreement with that observed in experiment, indicating that human listeners do have expectations about the source spectrum.

Much better agreement with the experimental data is obtained if the model assumes the human listener uses a prior on the SG of the source spectrum, as the polar error rate now follows a similar pattern as observed in experiment. The observer clearly benefits from the prior in case of either small or large ripple densities, as in these cases the error rate is considerably smaller than without prior (ISD). Yet, for values between [0.5–1.5] ripples/octave, including the prior results in a larger error rate compared to assuming no prior at all. This behavior can be attributed to the fact that, for these ripple densities, the stimulus used in the experiments does not fit the listener’s prior thereby confusing the localization process. This can be understood qualitatively from Fig. 2 where we show respectively the spectral magnitude for the stimuli with different ripple densities, their cochlear representations and the SG. If we inspect the standard deviation (std) of the SG, see Fig. 2d, we see a similar pattern as for the polar error rate: it starts low, increases, peaks, to decrease again and level off to a small std. From the covariance matrix we can infer that the listener expects a source with a SG std of $\approx 8.5$dB/ERB (mean of diagonal of covariance matrix), yet for ripple densities ranging between [0.75–3] ripples/octave, the source SG exceeds this value. Consequently the stimulus does not fit the prior and is too unexpected for the listener, which translates in a reduced localization performance.

**Fig. 6:**
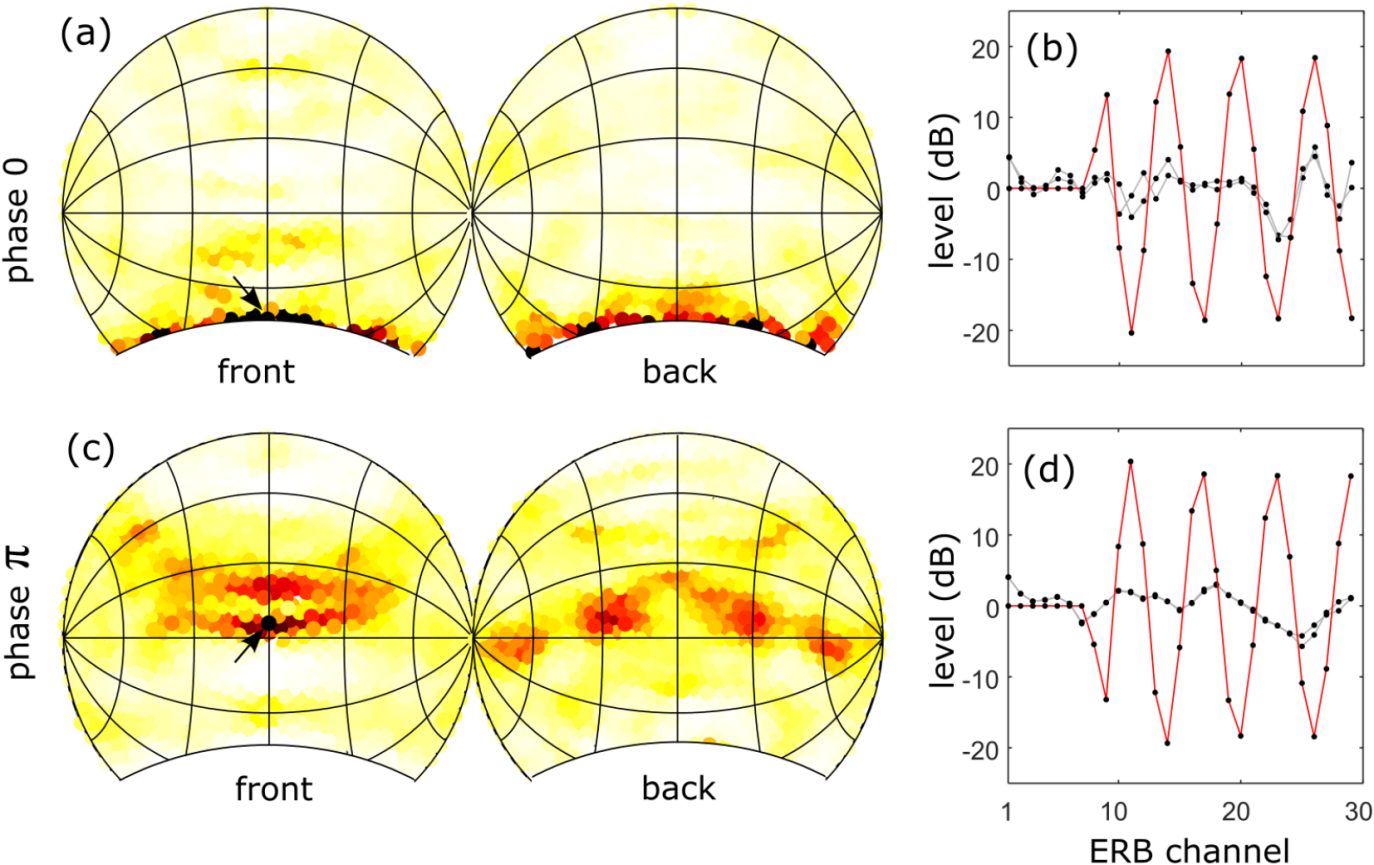
Comparing localization response behaviour in case of $\phi =0$ and $\pi$ (40db ripple depth,1 ripple/octave). (**a**, **c**) The response distribution in case of uniform stimulus presentation (arrow = direction with highest response value); (**b**, **d**) the attractor’s binaural SG template (black) and the ripple SG (red).

The same reasoning explains why the error rate increases with ripple magnitude: because the std of the SG also increases with increasing magnitude. It fails to explain though why the error rate depends on the ripple phase. This is rather unexpected, since the std of the SG is independent of the phase (except for small boundary effects). Indeed, the SG only differs in sign for phase 0 and $\pi$ and consequently the std of the SG is identical for both phases, see the red curves/dot in Fig. 2. A possible explanation could be that in case of $\phi =0$, the modulated source spectrum has a larger resemblance to one/some of the directional templates. This would cause confusion between spectral features due to the source spectrum and those due to the HRTF, resulting in an increased error rate.

To assess this hypothesis, we plot in Fig. 6 the distribution of simulated localization responses averaged over all different individuals (binaural SG model). From this distribution of responses, it is clear that not all directions are equally likely to be chosen, some ‘attract’ more responses than others. Moreover, this distribution depends on the phase: in case of $\phi =0$, one is much more likely to (wrongly) assign sources to directions in the lower part of the hemisphere, both in the front and the back, whereas in case of $\phi =\pi$ the response is biased more towards (slightly above) the horizontal plane. Interestingly, such a difference in bias is also observed in the experimental localization data presented by Macpherson et al.^31^ (see Fig. 10 in their manuscript, especially for subjects S64 and S77) and results in the difference in error rates. To understand the nature of this bias, we select for each response distribution the direction with the highest response (marked by an arrow in Fig. 6a,c). For these directions which act as ‘attractors’, we then plot their proper left and right SG templates, together with the SG of the ripple-spectrum source (Fig. 6b,d). In case of $\phi =0$, the attractor’s template (averaged over all individuals) is somewhat in phase with the source SG and this resemblance biases the responses towards the attractor. This is less so for $\phi =\pi$, where the similarity is less obvious.

The model using the SG consistently produces polar error rates which are too small compared to the experimental results (see Fig. 4). A possible explanation could be that the observer does not use all information available. We studied two such ‘suboptimal’ representations/feature sets, where either only the ipsilateral SG or only the positive SG is being processed. Both representations result in significantly larger errors, as expected, yet the model using the positive SG deviates also qualitatively from the experimentally observed error rates. Therefore, our model simulations contradict the hypothesis that the positive SG is used for sound localization. On the other hand, the experimentally observed localization performance lies mostly in between that predicted by the model using the full binaural SG and the model using only the ipsilateral SG. Hence, these results suggest that human listeners do indeed use binaural information, but assign more weight to the ipsilateral input. This is in agreement with previous findings that for increasing lateral angles, listeners increasingly rely on the ipsilateral spectral input rather than binaural input for polar angle estimation^36,37^.

Our results show that including a prior on the SG improves localization, at least if the source spectrum fits the prior. A more narrow prior allows for a more accurate localization, but only if the source matches the prior. This becomes clear if one uses a prior which is too narrow for the stimulus. e.g., as used in Reijniers et al. ^3^ for the case of an unchanging stimulus. When we calculate the error rates using this prior, the model fails to explain the observed error rates, both qualitatively and quantitatively, see Supplementary Information. Hence, an interesting question arises: do human listeners update their prior according to the stimuli they are confronted with? Our results provide indirect evidence that, at least in the context of sound localization experiments, human listeners, instead of smoothly adapting to the presented stimulus ensemble, use a generic, multi-purpose spectral prior based on a broad, ecologically valid stimulus set. Including such a prior in case the subject is repeatedly confronted with the same flat spectrum stimulus results in angular errors which are larger, compared to using the narrow prior, but resemble the true errors more closely (see Fig. 5). Nevertheless, the predicted errors are still smaller than the experimentally obtained errors, even when using the ipsilateral SG as input to the model. As conjectured before^3,28^, the remaining difference is at least partly due to the pointing error, i.e. the error introduced when the subject communicates the perceived source direction. The fact that, different from the human experiments, the simulated errors are not (substantially) larger in the rear than in the front, can also be explained by the pointing error, which may well increase with the reporting angle and hence be larger in the back^40^. Yet, it may also be due to the fact that we assumed that all signals received were well above hearing threshold and the environmental noise level. Indeed, it has been shown that localization performance decreases with increasing noise levels, localization in the back being most sensitive to noise^41^.

## Conclusion

In this paper, we have extended the previously proposed ideal-observer model for static sound localization of brief broadband stimuli, so that it can deal with source stimuli whose spectrum is not known to the listener. An ecologically valid prior of the source spectrum was derived from two databases. Although the databases were of a different nature (environmental sounds vs. human speech), both resulted in a similar covariance matrix in case of the SG representation, hence allowing for a single generic prior to be used when confronted with unknown sources. The model relies only on an HRTF database, noise parameters derived from psycho-acoustic discrimination experiments on ITD and ILD, and an ecologically valid database of sound stimuli. Hence, it uses no free parameters to fit the experimental data.

As input to this ideal-observer model, the binaural SG supplemented with the ILD was shown to contain the same information as the binaural spectral magnitude. This stimulus representation is more compact and has the additional advantage that the localization process can be separated in two parallel processes, one involving lateral angle localization based on binaural cues (ITD and ILD) and one involving polar angle estimation based on monaural cues, e.g. of the ipsilateral side, in agreement with observations from both psychoacoustic experiments and physiology.

The ideal-observer model using the SG as input was able to explain how the localization performance (polar error rate) changes as the stimulus spectrum (ripple characteristics) is varied between trials. The experimental performance was shown to lie in between predictions produced by the models using the binaural SG and the ipsilateral SG. This suggests that the listener uses more information than only the ipsilateral SG, yet less than the full binaural SG. This conclusion is again in agreement with previous findings that for increasing lateral angles, humans increasingly rely on ipsilateral rather than binaural spectral input for polar angle estimation. On the other hand, despite physiological indications that only the frequency channels with a positive SG are relayed for polar angle estimation, our model simulations do not support this hypothesis.

Finally, the ideal-observer model using the ecologically valid SG prior provided a better fit to the experimental data on localization of flat spectrum stimuli, compared to the model using a source-specific (narrow) prior. This suggests that human listeners may choose to use a multi-purpose rather than a situation-specific spectral prior, even if they are repeatedly confronted with the same stimulus spectrum. This conservative stance may be an evolutionary adapted strategy, where robustness, i.e. the ability to deal with unexpected stimuli, is preferred to localization accuracy. Yet, whether this spectral prior is being updated, and if so, at what rate and to what extent, is a question for future research.

## Supplementary Information

Supplementary Information.

## Data Availability

The datasets used and/or analysed during the current study are available from the corresponding author on request.
