# Supplementary material for: Ideal-observer model of human sound localization of sources with unknown spectrum
Source: Sci Rep. 2025 Mar 1;15:7289. doi: 10.1038/s41598-025-91001-3 (PMC11873210; doi:10.1038/s41598-025-91001-3)
Supplement: Supplementary file 1 — Supplementary Information. [file 41598_2025_91001_MOESM1_ESM.pdf]

Supplementary Information

Ideal-Observer Model of Human Sound Localization of Sources with Unknown Spectrum

J. Reijniers, G. McLachlan, B. Partoens, H. Peremans

1 Ideal-observer model

1.1 Bayesian inference

The observer receives an acoustic input $\mathbf{X}$ and wants to infer $P(\boldsymbol{\theta}|\mathbf{X})$, the probability that, given the observed acoustic input, the sound originates from direction $\boldsymbol{\theta}$. Using Bayes' rule, the prior $P(\boldsymbol{\theta})$ can then be updated to the posterior

$$P(\boldsymbol{\theta}|\mathbf{X}) = \frac{1}{C} P(\mathbf{X}|\boldsymbol{\theta}) P(\boldsymbol{\theta}) \tag{1}$$

with C a normalization constant such that $\int P(\boldsymbol{\theta}|\mathbf{X}) P(\boldsymbol{\theta}) d\boldsymbol{\theta} = 1$. There are some indications that human observers use a non-uniform spatial prior $P(\boldsymbol{\theta})$, with a bias towards the horizontal plane reflecting the higher probability/relevance of sources at these directions [4]. Yet, to keep the unknowns to a minimum, we consider a uniform prior probability.

1.2 Relevant acoustic input

It is not yet clear which acoustic input is relevant for sound localization, in fact, this is one of the aims of using an ideal-observer approach: to investigate which acoustic features contain sufficient information on the source direction.

Obviously, all acoustic information that is measured and relayed by the cochlea may be used and would be used by an ideal-observer. Yet, it is likely that this information is redundant and the brain only uses part of this information, the so-called acoustic features. A common assumption is that the interaural time difference (ITD) contains all phase information that is relevant for localization; all other information is contained in the binaural spectral magnitude. The acoustic information about the sound source position is then contained in the vector

$$\mathbf{X} = [X_{\mathrm{ITD}}, \mathbf{X}^l, \mathbf{X}^r], \tag{2}$$

i.e., the measured ITD supplemented with $\mathbf{X}^l$ and $\mathbf{X}^r$, representing the spectral magnitude, as measured by respectively the left and right ear. The $[\cdot, \cdot]$ operation represents the concatenation into one single vector.

1.2.1 Interaural Time Difference

The first cue we take into account is the ITD. The ITD as it is measured can be written as

$$X_{\mathrm{ITD}} = \mathrm{ITD}(\boldsymbol{\theta}) + \delta_{\mathrm{ITD}}, \tag{3}$$

with $\mathrm{ITD}(\boldsymbol{\theta})$ the true ITD corresponding to direction $\boldsymbol{\theta}$, and δ_{ITD} the error on the ITD measurement, due to the limited precision of the hearing apparatus and/or environmentally induced uncertainty (e.g air turbulence, etc).

1.2.2 Binaural Spectral Magnitude

The ITD is supplemented with the binaural spectral magnitude. As the hearing apparatus can be approximated to perform a logarithmic compression (see further), we consider the log-magnitude spectra in the left and the right ear. The latter are given by the sum of the log-magnitudes of the sound source spectrum $\mathbf{S}$ and the HRTF for the left and right ear, respectively $\mathbf{H}^l(\boldsymbol{\theta})$ and $\mathbf{H}^r(\boldsymbol{\theta})$.

$$\begin{aligned} \mathbf{X}^l &= \mathbf{S} + \mathbf{H}^l(\boldsymbol{\theta}) + \boldsymbol{\delta}^l \\ \mathbf{X}^r &= \mathbf{S} + \mathbf{H}^r(\boldsymbol{\theta}) + \boldsymbol{\delta}^r, \end{aligned} \quad (4)$$

with the respective errors $\boldsymbol{\delta}^l$ and $\boldsymbol{\delta}^r$ due to the limited precision of the hearing system in measuring the frequency content of the incoming sound stimulus. The symbols in bold font denote vectors with values along the frequency axis (at fixed frequencies, the so-called frequency channels, see further). The spectrum of the sound source $\mathbf{S}$ is often not known exactly by the listener. In the following, we assume $\mathbf{S}$ to be identical for both ears, i.e., we assume that the sound source is in the far field.

1.3 Modeling prior information used by the listener

In order to estimate the sound source direction, the listener does not solely rely on the acoustic information $\mathbf{X}$, but interprets these cues in the context of prior information defining a measurement model based on prior knowledge of the spectral filtering $\mathbf{H}^l(\boldsymbol{\theta})$ and $\mathbf{H}^r(\boldsymbol{\theta})$ as well as on prior information on the behavior of the respective errors $\boldsymbol{\delta}_{ITD}$, $\boldsymbol{\delta}^l$, $\boldsymbol{\delta}^r$ and prior information on the spectrum of the sound source $\mathbf{S}$. In the following we will discuss these a priori assumptions and propose quantitative models for them. We acknowledge that these models are simplifications leaving out many detailed observations from spatial hearing research. Nevertheless, this simplified model will allow us to reproduce and gain insight into essential properties of human sound localization as measured experimentally.

1.3.1 ITD noise prior

In order to extract sound source direction information from the ITD cues, the listener needs to have a noise model for the ITD measurement, δ_{ITD}, i.e. make (a priori) assumptions about the distribution of the error $P(\delta_{\mathrm{ITD}})$. This error is related to the precision of the hearing system in measuring ITD, which also depends on the frequency content and the intensity of the incoming sound [6]. We chose to estimate this error distribution based on the just-noticable difference (jnd) measure extracted from ITD discrimination experiments with clearly audible broadband noise stimuli, as these conditions correspond best with the human localization experiments we will be modeling. The jnd is defined as the smallest difference Δx between two cue values (x and $x + \Delta x$), that can be correctly detected for a specified percentage of the experiments. The results of ITD experiments using broadband stimuli [12] show that the jnd depends on the particular ITD value around which it is measured. In particular, these experiments show that human ITD discrimination performance is approximately a linear function of ITD, but with a nonzero offset at zero ITD,

$$\mathrm{jnd}(\mathrm{ITD}) = a + b \cdot \mathrm{ITD}, \quad (5)$$

with $a = 32.5\,\mu\mathrm{s}$ and $b = 0.095$.

In order to simplify the analysis, we would like to model the error δ_{ITD} by a normal distribution, with a variance σ_{ITD} which is *independent* of the ITD itself. Therefor, we transform the ITD scale expressed in time units into a new scale expressed in jnd units,

$$\mathrm{ITD} \rightarrow \int_0^{\mathrm{ITD}} \frac{1}{\mathrm{jnd}(x)} dx = \frac{1}{b}[\log(a + b \cdot \mathrm{ITD}) - \log(a)], \quad (6)$$

where $dx/\mathrm{jnd}(x)$ indicates the number of jnd's in the interval dx and we integrate over x to arrive at the total number of jnd's for a given ITD. After this transformation, the jnd obtained in the experiment can be related to the model parameter $\sigma_{\mathrm{ITD}} = 0.569$ jnd, see Supplementary Information of [13].

1.3.2 Directional filter information

In order to extract the spatial information encoded in the binaural spectrum, the listener should have information on the directional filtering performed by the head, ears and torso as summarized by $\mathbf{H}^l(\theta)$ and $\mathbf{H}^r(\theta)$. We will assume that learning provides humans with a very accurate model of their HRTF. If the listener is suddenly confronted with a different HRTF, e.g. by use of artificial pinnae or by use of a different HRTF in a virtual sound localization experiment, localization performance is degraded until the internal model is adapted to include the new HRTF [9, 15].

1.3.3 Spectral noise prior

The precision of the measurement of the spectrum is limited by the resolution of the hearing system along both the frequency axis and the intensity axis. In the following we include this in the model (1) by choosing discrete independent frequency channels and (2) by assessing the internal noise distribution in each of the frequency channels. This is done by modelling basic properties of the hearing system, as determined through psychoacoustic experiments in earlier studies.

The spectral representation along the frequency axis is determined by the neural transduction of the movement of the basilar membrane. As a consequence, the spectrum $\mathbf{X}$ can be represented (without loss of information) as a vector representing the responses of a limited number of frequency channels. To choose these so-called frequency channels, we consider frequencies separated by the equivalent rectangular bandwidth (ERB) of the auditory filter, as these frequencies channels are approximately independent. According to Moore and Glasberg [5], the ERB (in Hz) is function of the center frequency f (in kHz) according to

$$\mathrm{ERB}(f) = 24.7\mathrm{Hz}\left(\frac{4.37f}{1000\mathrm{Hz}+1}\right). \tag{7}$$

We choose the frequency channels such that their bandwidths correspond to the ERB and the corresponding bandpass filters are adjacent but do not overlap. The first frequency channel is at 300 Hz, and has bandwidth $\mathrm{ERB}(f = 0.3\mathrm{kHz}) = 57.08\mathrm{Hz}$. The next frequency channel is at $f = 357,08\mathrm{Hz}$, and so on. Hence, to cover the frequency range [300Hz-12kHz], we arrive at $n_f = 30$ frequency channels with non-overlapping rectangular bandwidths.

We assume that the intensity of the incoming sound is above the hearing threshold, i.e. in each of the frequency channels the intensity is above the noise level. The measurement of the log-magnitude is independent in each of the frequency channels (by choice of ERB channels), and the precision in each of the channels is determined by $\boldsymbol{\delta}^l$ and $\boldsymbol{\delta}^r$, i.e. by the error on the measurement of the left and right spectrum. As was the case for the ITD error, through experience the listener assesses the precision of his hearing system and hence learns the error distributions $\boldsymbol{\delta}^l$ and $\boldsymbol{\delta}^r$. We consider the log-magnitude (instead of the magnitude) as psycho-acoustic experiments have shown that magnitude jnd scales linearly with the magnitude strength. Therefore, if we want to model the spectral noise δ_S as additive noise with a variance σ_S *independent* of the magnitude strength, we should consider the log-magnitude. As a consequence, we can model the error on the log-magnitude spectrum due to internal noise as a multivariate normal

distribution, given by

$$\begin{aligned} P(\boldsymbol{\delta}^l) &\sim \mathcal{N}(0, \boldsymbol{\Sigma}^l) \\ P(\boldsymbol{\delta}^r) &\sim \mathcal{N}(0, \boldsymbol{\Sigma}^r), \end{aligned} \tag{8}$$

with $\boldsymbol{\Sigma}^l = \boldsymbol{\Sigma}^r$ the monaural covariance matrices. Because of the choice of non-overlapping frequency channels, we assume each of the frequency channels to operate independent of the others, i.e. the covariance matrices are diagonal. Moreover, if we assume that the error distribution is identical in each of the channels, i.e., a normal distribution with variance σ_ϵ^2 , then the covariance matrices read $\boldsymbol{\Sigma}^l = \boldsymbol{\Sigma}^r = \sigma_\epsilon^2 \cdot \mathbf{I}$, where $\mathbf{I}$ is the unity matrix with order $n_f \times n_f$.

In order to estimate the parameter of this error distribution σ_ϵ, we again turn to psychoacoustic experiments that tested for intensity discrimination of broad band noise [7], see Supplementary information of [13]. For our model, using the frequency channels as described above, this results in $\sigma_\epsilon = 3.5\text{dB}$.

1.3.4 Source prior

With the above transformations, we have established that, given a particular source spectrum $\mathbf{S}$ and source direction $\boldsymbol{\theta}$, the likelihood of measuring an acoustic input $\mathbf{X}$, $P(\mathbf{X}|\boldsymbol{\theta}, \mathbf{S})$, can be modeled as a multivariate normal distribution. Yet, the observer does not necessarily know the source spectrum. An ideal-observer would handle this by use of a prior on the source spectrum $P(\mathbf{S})$. Hence, our model assumes [13] that the listener has some knowledge of the source spectrum $\mathbf{S}$, i.e. the source has an expected spectrum $\hat{\mathbf{S}}$ with an uncertainty that is captured by the source covariance matrix $\boldsymbol{\Sigma}_S$:

$$P(\mathbf{S}) = \mathcal{N}(\hat{\mathbf{S}}, \boldsymbol{\Sigma}_S). \tag{9}$$

Note that this assumes that the observer's prior belief on the source spectrum can be modeled as a multivariate normal distribution.

1.4 Bayesian inference revisited

Assuming the prior $P(\mathbf{S})$, an ideal-observer would integrate over all possible source spectra to calculate the likelihood, i.e.,

$$P(\mathbf{X}|\boldsymbol{\theta}) = \int_S P(\mathbf{X}|\boldsymbol{\theta}, \mathbf{S}) P(\mathbf{S}) d\mathbf{S}. \tag{10}$$

Since both $P(\mathbf{X}|\boldsymbol{\theta}, \mathbf{S})$ and $P(\mathbf{S})$ are multivariate normal distributions, it can be shown that this integral simplifies to

$$P(\mathbf{X}|\boldsymbol{\theta}) = \mathcal{N}(\mathbf{T}(\boldsymbol{\theta}), \boldsymbol{\Sigma}), \tag{11}$$

i.e., a multivariate normal distribution centred around the angular template

$$\mathbf{T}(\boldsymbol{\theta}) = [\text{ITD}(\boldsymbol{\theta}), \mathbf{H}^l(\boldsymbol{\theta}) + \hat{\mathbf{S}}, \mathbf{H}^r(\boldsymbol{\theta}) + \hat{\mathbf{S}}], \tag{12}$$

with covariance matrix

$$\boldsymbol{\Sigma} = \begin{bmatrix} \sigma_{\text{ITD}}^2 & 0 & 0 \\ 0 & \boldsymbol{\Sigma}_\epsilon + \boldsymbol{\Sigma}_S & \boldsymbol{\Sigma}_S \\ 0 & \boldsymbol{\Sigma}_S & \boldsymbol{\Sigma}_\epsilon + \boldsymbol{\Sigma}_S \end{bmatrix}, \tag{13}$$

where $\mathbf{H}^l(\boldsymbol{\theta})$ and $\mathbf{H}^r(\boldsymbol{\theta})$ represent the left and right ear directional filtering as described by the HRTF, σ_{ITD}^2 and $\boldsymbol{\Sigma}_\epsilon = \sigma_\epsilon^2 \mathbf{I}$ are the error variance and covariance matrix respectively describing the measurement noise and $\hat{\mathbf{S}}$ and $\boldsymbol{\Sigma}_S$ define the observer's prior on the source spectrum. Note that the template $\mathbf{T}(\boldsymbol{\theta})$ contains the sum of the HRTF $\mathbf{H}(\boldsymbol{\theta})$ and the expected spectral log-magnitude $\hat{\mathbf{S}}$. With regards to the covariance matrix $\boldsymbol{\Sigma}_S$, note that the left and right source spectra are considered identical (fully correlated).

1.5 Alternate spectral features

The ideal-observer model described above assumes optimal use of the binaural spectral magnitude information, hence both left and right log-magnitude spectra are used and have equal weight in the estimation process, independent of source direction. As mentioned in the introduction, there is evidence that either of these assumptions might not be true for the human sound localization process. Below, we describe how we adapt the inputs to the ideal-observer model so as to better correspond with the assumptions about the human localization process that have been suggested as alternatives.

1.5.1 Binaural Spectral Gradient

Instead of the acoustic input shown in Eq. 2, we consider the following input:

$$\mathbf{X} = [X_{\mathrm{ITD}}, X_{\mathrm{ILD}}, \mathbf{dX}^l, \mathbf{dX}^r], \tag{14}$$

i.e., the ITD is supplemented with the broadband ILD, here defined as

$$X_{\mathrm{ILD}} = \frac{1}{N}\sum_{i=1}^{N}(\mathbf{X}_i^l - \mathbf{X}_i^r). \tag{15}$$

and the left and right SGs which are defined as the set of differences between neighbouring frequency channels

$$\mathbf{dX}_i^{l/r} = \mathbf{X}_i^{l/r} - \mathbf{X}_{i-1}^{l/r}, \tag{16}$$

Since this transformation from spectral magnitude to SG is in fact a linear transformation

$$\begin{bmatrix} \mathbf{dX}_1^l \\ \mathbf{dX}_2^l \\ \vdots \\ \mathbf{dX}_{n-1}^r \end{bmatrix} = \underbrace{\begin{bmatrix} 1 & -1 & 0 & \cdots & 0 \\ 0 & 1 & -1 & \cdots & 0 \\ 0 & 0 & 1 & \cdots & 0 \\ \vdots & \vdots & \vdots & \vdots & \vdots \\ 0 & 0 & 0 & \cdots & -1 \end{bmatrix}}_{\mathbf{M}} \cdot \begin{bmatrix} \mathbf{X}_1^l \\ \mathbf{X}_2^l \\ \mathbf{X}_3^l \\ \vdots \\ \mathbf{X}_n^l \end{bmatrix}, \tag{17}$$

the likelihood function has the same functional form as in Eq. 11, but with a different angular template

$$\mathbf{T}(\boldsymbol{\theta}) = [\mathrm{ITD}(\boldsymbol{\theta}), \mathrm{ILD}(\boldsymbol{\theta}), \mathbf{dH}^l(\boldsymbol{\theta}) + \hat{\mathbf{dS}}, \mathbf{dH}^r(\boldsymbol{\theta}) + \hat{\mathbf{dS}}], \tag{18}$$

and covariance matrix

$$\boldsymbol{\Sigma} = \begin{bmatrix} \sigma_{\mathrm{ITD}}^2 & 0 & 0 & 0 \\ 0 & 2\sigma_\epsilon^2/N & 0 & 0 \\ 0 & 0 & \boldsymbol{\Sigma}_{d\epsilon} + \boldsymbol{\Sigma}_{dS} & \boldsymbol{\Sigma}_{dS} \\ 0 & 0 & \boldsymbol{\Sigma}_{dS} & \boldsymbol{\Sigma}_{d\epsilon} + \boldsymbol{\Sigma}_{dS} \end{bmatrix}. \tag{19}$$

The corresponding covariance matrices $\boldsymbol{\Sigma}_{d\epsilon}$ and $\boldsymbol{\Sigma}_{dS}$ and the expected SG $\hat{\mathbf{dS}}$ can be calculated using the transformation matrix $\mathbf{M}$ as defined in Eq. 17:

$$\boldsymbol{\Sigma}_{d\epsilon} = \mathbf{M}\boldsymbol{\Sigma}_\epsilon\mathbf{M}^T,\ \boldsymbol{\Sigma}_{dS} = \mathbf{M}\boldsymbol{\Sigma}_S\mathbf{M}^T,\ \hat{\mathbf{dS}} = \mathbf{M}\hat{\mathbf{S}}. \tag{20}$$

This representation contains exactly the same directional information as the binaural log-magnitude representation Eq. 2. Indeed, if the SG representation from Eq. 14 were to be supplemented with

$$X_{\mathrm{SL}} = \frac{1}{2N}\sum_{i=1}^{N}(\mathbf{X}_i^l + \mathbf{X}_i^r), \tag{21}$$

which is a measure of the sound level (SL), this representation can be derived from the other (Eq. 2) and vice versa, proving that they contain the same information. As the overall sound level carries no directional information, the SG representation contains all the relevant information.

1.5.2 Positive Spectral Gradient

Here, we consider the case where only the positive SG values [2] are used by the direction estimation process (supplemented with ITD and ILD). While there are indications that only the positive SG is relayed to the dorsal cochlear nucleus for polar angle estimation [14], the evidence is not very strong, since this was not observed in guinea pigs [16]. Note that in this case there is a loss of information as this transformation of the log-magnitude spectra can not be inverted.

We consider the same information as in Eq. 14, but to simplify the derivation, we define $\mathbf{dX} = [\mathbf{dX}^l, \mathbf{dX}^r]$, i.e., we merge the binaural SG into one single vector. Suppose that the positive SG values are at indices p and the negative ones at indices n. If only the positive gradient values $\mathbf{dX}_p$ are used for localization, then the likelihood of obtaining these particular positive gradient values (and an unspecified negative gradient value elsewhere) can be written as

$$P(\mathbf{dX}_p, \mathbf{dX}_n < 0|\boldsymbol{\theta}). \tag{22}$$

Note that this likelihood does include the information that the SG vector was negative for indices n. Indeed, the exact values of these negative gradients may not be relayed for higher processing, yet the absence of a positive gradient informs the listener's brain that the gradient was negative and thus also carries information. Therefore, one should integrate over all possible ways the gradient could be negative in these channels, resulting in

$$\begin{aligned} & P(\mathbf{dX}_p, \mathbf{dX}_n < 0|\boldsymbol{\theta}) \\ &= \int_{-\infty}^{0} P(\mathbf{dX}_p, \mathbf{dX}_n|\boldsymbol{\theta}) d\mathbf{dX}_n \\ &= P(\mathbf{dX}_p|\boldsymbol{\theta}) \int_{-\infty}^{0} P(\mathbf{dX}_n|\boldsymbol{\theta}, \mathbf{dX}_p) d\mathbf{dX}_n \\ &\approx P(\mathbf{dX}_p|\boldsymbol{\theta}) \int_{-\infty}^{0} P(\mathbf{dX}_n|\boldsymbol{\theta}) d\mathbf{dX}_n. \end{aligned} \tag{23}$$

The last step is an approximation, since possible statistical dependencies between any of the values $\mathbf{dX}_p$ and $\mathbf{dX}_n$ given a hypothesized target direction θ are neglected. Using this expression, the likelihood of Eq. 11 then changes to

$$P(\mathbf{X}|\boldsymbol{\theta}) \sim \mathcal{N}(\mathbf{T}^+(\boldsymbol{\theta}), \boldsymbol{\Sigma}^+) \times \boldsymbol{\Phi}(0; \mathbf{T}^-(\boldsymbol{\theta}), \boldsymbol{\Sigma}^-) \tag{24}$$

with $\boldsymbol{\Phi}$ the multivariate normal cumulative distribution function and

$$\begin{aligned} \mathbf{T}^+(\boldsymbol{\theta}) &= [\mathrm{ITD}, \mathrm{ILD}, \mathbf{dH}_p + \hat{\mathbf{dS}}_p](\boldsymbol{\theta}), \\ \mathbf{T}^-(\boldsymbol{\theta}) &= [\mathbf{dH}_n + \hat{\mathbf{dS}}_n](\boldsymbol{\theta}), \end{aligned} \tag{25}$$

with $\boldsymbol{\Sigma}^+$ and $\boldsymbol{\Sigma}^-$ the corresponding subsampled versions of the covariance matrix $\boldsymbol{\Sigma}$. Note that p and n are determined only by the sign of the gradient of the measured input (and *not* by the sign of $\mathbf{dH}$).

Ipsilateral Spectral Gradient

Here, we consider another transformation of the SG that also results in information loss, i.e. we limit the spectral input to the SG of the presumed ipsilateral ear. Again there are a number of studies that demonstrated that the spectral cues from the ear contralateral to the target have a decreasing weight in the direction estimation process becoming negligible for targets sufficiently displaced from the midline [8, 11]. Considering only the ipsilateral SG can be viewed as a limiting case. The true performance should be somewhere in between that obtained using the binaural SG (upper limit) and that obtained with the ipsilateral SG (lower limit). For simplicity, we base the decision on which SG is used as input to the model, $\mathbf{dX}^l$ or $\mathbf{dX}^r$, solely on the sign of the ITD, i.e.,

$$\begin{aligned} \mathbf{X} &= [X_{\text{ITD}}, X_{\text{ILD}}, \mathbf{dX}^l] \qquad && \text{if } X_{\text{ITD}} > 0, \\ &= [X_{\text{ITD}}, X_{\text{ILD}}, \mathbf{dX}^r] && \text{if } X_{\text{ITD}} < 0, \end{aligned} \tag{26}$$

The corresponding templates and covariance matrix can be derived directly as the relevant subset of those listed in Eqs. 18 and 19. Since only a single monaural SG is considered, the correlation between left and right $\mathbf{dS}$ can no longer be exploited. Note, though, that the source spectrum correlation is still present and possibly exploited through the ILD. Hence, both the ITD and ILD remain truly binaural cues.

Interaural Spectral Difference

As a baseline, we also consider the case when the observer assumes no prior knowledge on the source spectrum at all, i.e., every source spectrum is equally probable. This situation is identical to using

$$\mathbf{X} = [X_{\text{ITD}}, \mathbf{X}^l - \mathbf{X}^r], \tag{27}$$

i.e., the ITD supplemented with the Interaural Spectral Difference (ISD) between left and right log-magnitude spectra, as already discussed in [13]. Indeed, since both ears experience an identical source spectrum, subtraction of both spectra removes the source spectrum from the representation, and hence this representation only contains information that is independent of the source spectrum, i.e.

$$\mathbf{T}(\boldsymbol{\theta}) = [\text{ITD}, \mathbf{H}^l - \mathbf{H}^r](\boldsymbol{\theta}), \tag{28}$$

and covariance matrix

$$\boldsymbol{\Sigma} = \begin{bmatrix} \sigma^2_{\text{ITD}} & 0 \\ 0 & 2\boldsymbol{\Sigma}_\epsilon \end{bmatrix}, \tag{29}$$

2 Results using an *ad hoc* narrow prior

In Ref. [13] we tried to explain localization performance in case the subjects are always confronted with a flat spectrum stimulus, of which only the sound level could vary between trials. All model parameters were derived from psycho-acoustic discrimination experiments, except for the prior on the source spectrum. We considered an *ad hoc* prior for the spectral magnitude of the source, which was thought to be appropriate for the listener in that particular experimental setting: we devised a rather narrow prior centered on a flat expected spectrum (variance of $\sigma_S^2 = (3.5\text{dB})^2$ on the diagonal of $\boldsymbol{\Sigma}_S$); the covariance between channels i and j was set to $\sigma_{ij}^2 = (5\text{dB})^2$ reflecting the uncertainty due to the variable sound level, see Ref. [13] for more information.

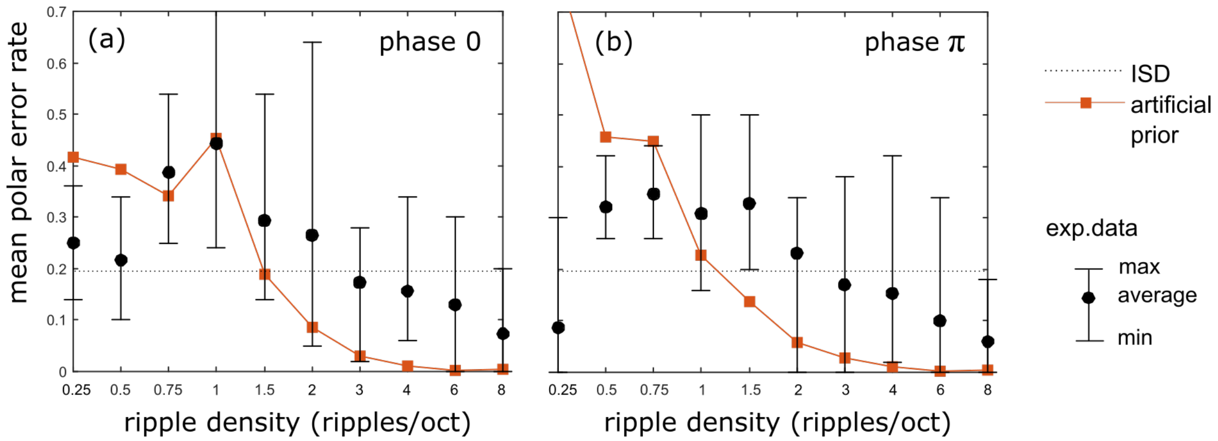

Figure S1: The average polar error rate as function of ripple density in case of phase 0 and phase π (40dB ripple depth) for the ideal-observer model presented in Ref. [13]

Now, if we were to use the proposed ideal-observer model with this prior on the source spectrum to localize sources with the rippled stimuli, as presented by Macpherson and Middlebrooks [10], then we obtain the results shown in Fig. S1. Here, we show the average polar error rates as function of ripple densities. We make the following observations: first, for small ripple densities, the polar error rates are much larger than observed experimentally, both in case of phase 0 and π. However, for large ripple densities, the polar error rate decreases to levels which are now much lower than seen in experiment. Hence, using a prior which assumes that the listener somehow takes advantage of the fact that the spectrum of the stimulus does not change between trials (except for level), does not reproduce the behavior seen in the experiment. Including the ecologically valid prior clearly results in much better agreement, both quantitatively and qualitatively, as is clear from Fig. 4 in the manuscript.

This behaviour of the polar error rate can again be understood from Fig. 2. Due to the choice of the narrow prior, the subject expects a rather flat source spectrum ($\sigma_S = (3.5dB)$), yet for low ripple densities, the spectral magnitude of the (manipulated) source spectrum has a fairly large variation of 40dB (bottom curve of Fig. 2(b)), which does not fit the prior and consequently this results in a large polar error rate. For higher ripple densities, though, the period of the ripple (or multiple periods) will fit within a single ERB band, and consequently the spectrum variations are averaged out. As this flattened spectrum (see top curve of Fig. 2(b)) now fits the prior rather well, this results in low polar error rates.

Note that these simulation results contradict the results by Barumerli *et al.* shown in Fig. 8 of Ref. [1]. They ran similar simulations with the ideal-observer model from Ref. [13] (as implemented in the AMT toolbox) but obtained polar error rates which were very different from those shown above: polar errors were consistently very small, irrespective of ripple density. We can neither reproduce nor explain their results.

3 Results using CREMA prior

In Fig. S2 we show the mean polar error rate in case the CREMA prior is used. Note that the results are very similar to those when using the ESC-50 prior, see Fig. 4 in the manuscript.

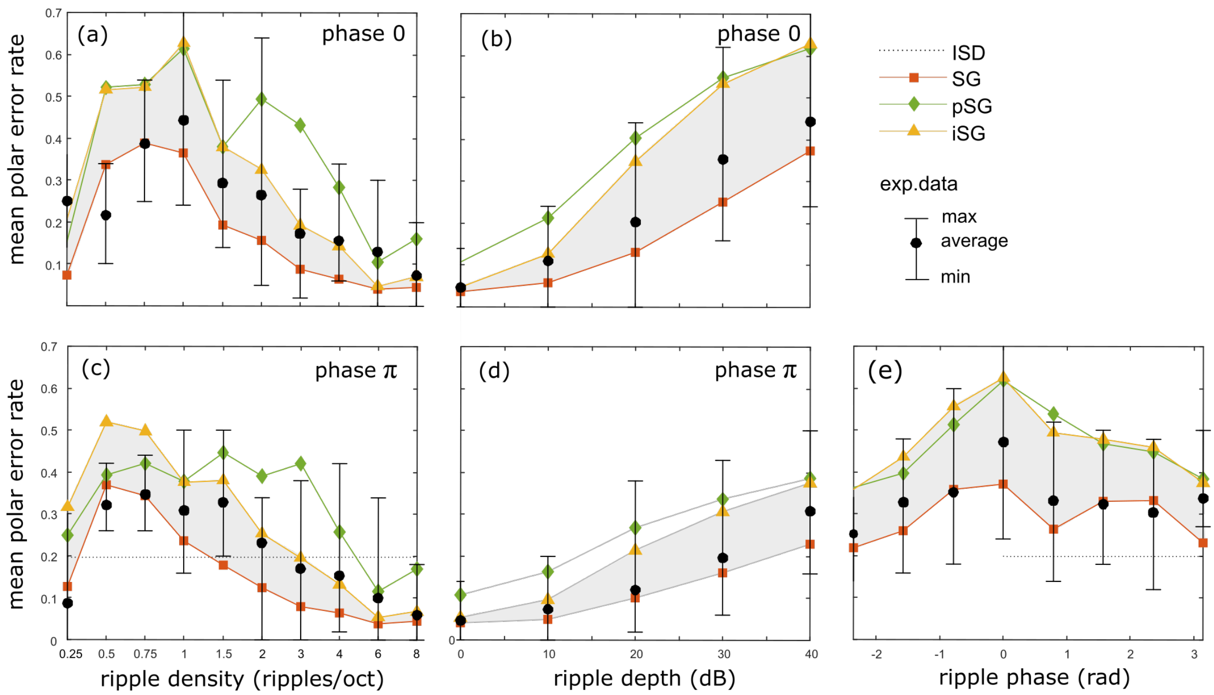

Figure S2: The same results as shown in Fig. 4, but in case the CREMA [3] prior is used.

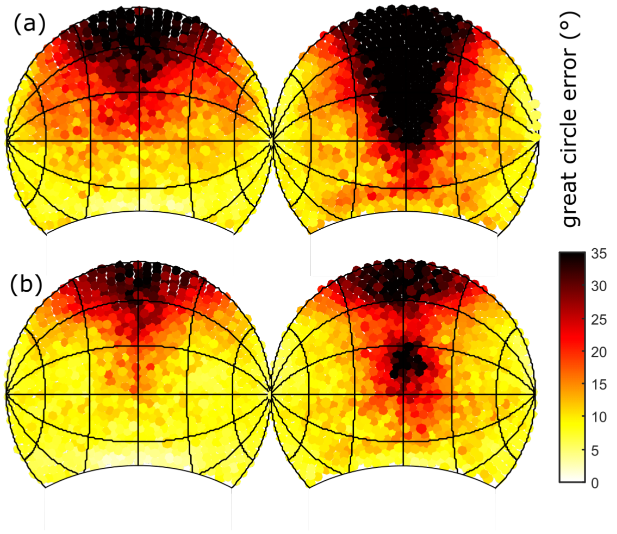

Figure S3: The great circle error for localization of a flat source stimulus as predicted by the ideal-observer model assuming the positive SG and the ESC-50 prior, (a) when the factor $\mathbf{\Phi}(0; \mathbf{T}^{-}(\boldsymbol{\theta}), \mathbf{\Sigma}^{-})$ of Eq. 24 was excluded and (b) when this factor was included.

4 Impact of omitting negative Spectral Gradient values altogether

In section 1.5.2 of this SI, we have derived the likelihood function when only the positive SG are used for direction estimation. We argued that an ideal-observer would also use the information that the SG vector was negative for some frequency channels resulting in an extra factor $\mathbf{\Phi}(0; \mathbf{T}^{-}(\boldsymbol{\theta}), \mathbf{\Sigma}^{-})$ in Eq. (24). As this factor was omitted in previous models considering the positive SG (e.g. in the model by Barumerli *et al.* [1]), we have investigated the importance of including this factor. Fig. S3(a) shows the great circle error in case the factor $\mathbf{\Phi}(0; \mathbf{T}^{-}(\boldsymbol{\theta}), \mathbf{\Sigma}^{-})$ in Eq. 24 was omitted, whereas in Fig. S3(b) it was included (as in the manuscript). It is clear that there is considerable directional information in knowing which frequency channels show an 'absence of a positive SG'. Hence, as including this information reduces the angular error significantly, an ideal-observer would make use of it.

References

[1] Roberto Barumerli, Piotr Majdak, Michele Geronazzo, David Meijer, Federico Avanzini, and Robert Baumgartner. A bayesian model for human directional localization of broadband static sound sources. *Acta Acustica*, 7:12, 2023.

[2] Robert Baumgartner, Piotr Majdak, and Bernhard Laback. Modeling sound-source localization in sagittal planes for human listeners. *The Journal of the Acoustical Society of America*, 136(2):791–802, 2014.

[3] Houwei Cao, David G Cooper, Michael K Keutmann, Ruben C Gur, Ani Nenkova, and Ragini Verma. Crema-d: Crowd-sourced emotional multimodal actors dataset. *IEEE transactions on affective computing*, 5(4):377–390, 2014.

[4] Rachel Ege, A John Van Opstal, and Marc M Van Wanrooij. Accuracy-precision trade-off in human sound localisation. *Scientific reports*, 8(1):16399, 2018.

[5] Brian R Glasberg and Brian CJ Moore. Derivation of auditory filter shapes from notched-noise data. *Hearing research*, 47(1-2):103–138, 1990.

[6] ER Hafter and J De Maio. Difference thresholds for interaural delay. *The Journal of the Acoustical Society of America*, 57(1):181–187, 1975.

[7] William M Hartmann and Zachary A Constan. Interaural level differences and the level-meter model. *The Journal of the Acoustical Society of America*, 112(3):1037–1045, 2002.

[8] P Hofman and A Van Opstal. Binaural weighting of pinna cues in human sound localization. *Experimental brain research*, 148:458–470, 2003.

[9] Paul M Hofman, Jos GA Van Riswick, and A John Van Opstal. Relearning sound localization with new ears. *Nature neuroscience*, 1(5):417–421, 1998.

[10] Ewan A Macpherson and John C Middlebrooks. Vertical-plane sound localization probed with ripple-spectrum noise. *The Journal of the Acoustical Society of America*, 114(1):430–445, 2003.

[11] Ewan A Macpherson and Andrew T Sabin. Binaural weighting of monaural spectral cues for sound localization. *The Journal of the Acoustical Society of America*, 121(6):3677–3688, 2007.

[12] Jennifer E Mossop and John F Culling. Lateralization of large interaural delays. *The Journal of the Acoustical Society of America*, 104(3):1574–1579, 1998.

[13] Jonas Reijniers, Dieter Vanderelst, Craig Jin, Simon Carlile, and Herbert Peremans. An ideal-observer model of human sound localization. *Biological cybernetics*, 108:169–181, 2014.

[14] Lina AJ Reiss and Eric D Young. Spectral edge sensitivity in neural circuits of the dorsal cochlear nucleus. *Journal of Neuroscience*, 25(14):3680–3691, 2005.

[15] Marc M Van Wanrooij and A John Van Opstal. Relearning sound localization with a new ear. *Journal of Neuroscience*, 25(22):5413–5424, 2005.

[16] Calvin Wu and Susan E Shore. Multisensory activation of ventral cochlear nucleus d-stellate cells modulates dorsal cochlear nucleus principal cell spatial coding. *The Journal of Physiology*, 596(18):4537–4548, 2018.
